# Developing a new perspective to study the health of survivors of Sichuan earthquakes in China: a study on the effect of post-earthquake rescue policies on survivors’ health-related quality of life

**DOI:** 10.1186/1478-4505-11-41

**Published:** 2013-10-29

**Authors:** Ying Liang, Xiukun Wang

**Affiliations:** 1 Department of Social Work and Social Policy, School of Social and Behavioral Sciences, Nanjing University, Nanjing 210023, People’s Republic of China; 2 School of Statistics and Mathematics, Zhejiang Gongshang University, Zhejiang 310018, People’s Republic of China

**Keywords:** Health-related quality of life, Post-earthquake rescue policies, SEM, SF-36, Survivors

## Abstract

**Background:**

Sichuan is a province in China with an extensive history of earthquakes. Recent earthquakes, including the Lushan earthquake in 2013, have resulted in thousands of people losing their homes and their families. However, there is a research gap on the efficiency of government support policies. Therefore, this study develops a new perspective to study the health of earthquake survivors, based on the effect of post-earthquake rescue policies on health-related quality of life (HRQOL) of survivors of the Sichuan earthquake.

**Methods:**

This study uses data from a survey conducted in five hard-hit counties (Wenchuan, Qingchuan, Mianzhu, Lushan, and Dujiangyan) in Sichuan in 2013. A total of 2,000 questionnaires were distributed, and 1,672 were returned; the response rate was 83.6%.

**Results:**

Results of the rescue policies scale and Medical Outcomes Study Short Form 36 (SF-36) scale passed the reliability test. The confirmatory factor analysis model showed that the physical component summary (PCS) directly affected the mental component summary (MCS). The results of structural equation model regarding the effects of rescue policies on HRQOL showed that the path coefficients of six policies (education, orphans, employment, poverty, legal, and social rescue policies) to the PCS of survivors were all positive and passed the test of significance. Finally, although only the path coefficient of the educational rescue policy to the MCS of survivors was positive and passed the test of significance, the other five policies affected the MCS indirectly through the PCS.

**Conclusions:**

The general HRQOL of survivors is not ideal; the survivors showed a low satisfaction with the post-earthquake rescue policies. Further, the six post-earthquake rescue policies significantly improved the HRQOL of survivors and directly affected the promotion of the PCS of survivors. Aside from the educational rescue policy, all other policies affected the MCS indirectly through the PCS. This finding indicates relatively large differences in the effects of different post-earthquake rescue policies on the HRQOL of survivors.

## Background

**Figure 1:**
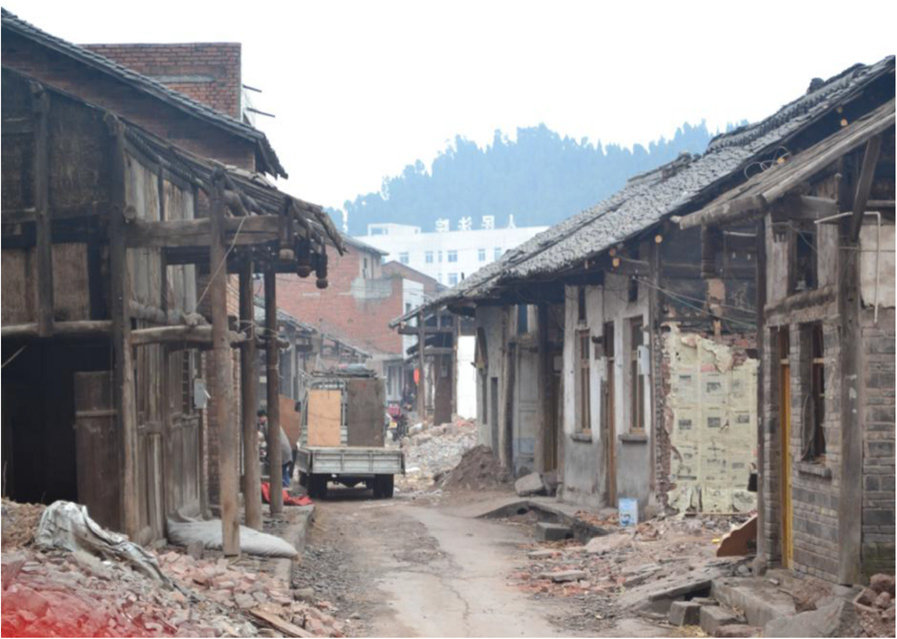
Collapsed houses in Guangyuan after the earthquake.

Sichuan is a province in China that has experienced many devastating earthquakes, thus increasing the suffering of its residents. On 12 May 2008, an earthquake with a magnitude of 8.0 on the Richter scale hit Wenchuan. This earthquake was the most devastating since the founding of New China. The earthquake also resulted in 5,335 students presumed dead or missing, more than 7,000 people becoming disabled, 152 million losing their jobs and lands, and 1,449 victims becoming lone people (orphans, elderly, and disabled people) [1]. On 30 June 2009, a 5.6 earthquake hit Mianzhu, Deyang, Sichuan. Two hours after the earthquake, aftershocks of 3.3 and 3.7 on the Richter scale occurred. Previous to these, more than 57,000 aftershocks had occurred in Sichuan with strengths of 4.0 to 6.4 on the Richter scale [2]. On May 2010, 11 earthquakes above 3.0 hit Sichuan, the largest with a strength of 5.0 [3]. On 20 April 2013, a severe 7.0 earthquake hit Lushan, Ya’an, resulting in 193 deaths, 12,211 injured, and more than 1,990,000 people affected [4]. Thus, frequent earthquakes have caused loss of lives, property, and economic-social development, and survivors in post-disaster areas are in need of assistance to restore normal production and improve their living conditions (Figure 1)^a^.

Post-disaster reconstruction has become the government’s top priority task. On 23 September 2008, less than half a year after the Wenchuan earthquake, the General Office of the State Council released the document “*National Master Plan on Post-Disaster Restoration and Reconstruction of Wenchuan Earthquake.*” The plan proposed 1 trillion yuan for the completion of reconstruction of hard-hit areas in Sichuan, Gansu, and Shaanxi within three years. The plan aims to make the basic living conditions and economic social development levels of survivors reach or exceed pre-disaster levels. The plan covers fiscal, tax, financial, land, industrial, and aid policies and counterpart support. For education, employment, poverty alleviation, and other aspects that urgently needed protection and support from the government, the plan particularly proposed rescue policies in education, orphans, employment, poverty, legal, and social security [5].

However, specific studies regarding post-earthquake rescue policies are weak and there is a research gap on the effect of government support policies. Recovery and reconstruction comprise a complicated systematic project, which can be divided into transition and overall periods [6]. First, several studies have focused on the performance of the government in emergency rescue during the transition period. Generally, the speed and quality of the government initiated and directed rescue were relatively high. The outcome of the rescue depended on a clear plan, effective coordination, cooperation, and support of all the participants [7]. Compared with the outcome of the Tangshan earthquake in 1976, the economic development of China has significantly improved the government’s ability to respond to natural disasters [8]. Second, the perspectives for studies on post-disaster reconstruction are diverse. On the one hand, many studies have focused on the interaction between the government and non-governmental organizations during the reconstruction; both the government and non-governmental organizations contributed [9,10] and the reconstruction was successful. The government allowed non-governmental organizations to participate in the reconstruction and was also more inclined to create a moderate policy-based environment [11,12]. On the other hand, some studies have tried to provide suggestions concerning reconstruction. Advanced information and communication technologies played an intermediary role in transmitting information during a disaster [13]. Thus, the openness and transparency of information help the government win the support of the people during reconstruction [14,15]. The establishment of an easily operated and widely covered disaster rescue model also helped the local government gain the survivor’s trust survivors [16]. Other studies have focused on the evaluation of the effect of specific government policies. One study assessed the implementation effect of the fertility assistance program that aimed to help couples achieve their reproductive desires. They found women were currently pregnant under the assistance program [17]. For the protection of forests, there was an increase in forest coverage rate since the enactment of the conservation programs [18]. However, to date, no studies have been conducted concerning the effects of the implemented rescue policies aiming to provide social and livelihood security.

The health condition of earthquake survivors is a controversial issue. However, many studies on survivors’ health have focused on the prevalence rate of mental disorders and their risk factors. It was found that 22.6% of the adolescent respondents were depressed, whereas 10.6% were reported to have suicidal ideation one month after the earthquake [19]. Three months after the earthquake, people who lived in temporary shelters or lost their families were more likely to report health problems [20]. Post-traumatic stress disorder (PTSD) also caused concerns. The important risk factors of PTSD include gender (female), age (elderly), and guilt regarding death or injury [21,22]; losing a child was also a significant predictor of mental problems [23]. Nevertheless, few studies have assessed the survivors’ health from the perspective of health-related quality of life (HRQOL).

Studies assessing HRQOL include one focused on children, indicating that the HRQOL of children with PTSD rapidly declined [24]. Therefore, mental health improvement can enhance their HRQOL [25]. Most of the HRQOL studies have focused on the positive effect of social support on survivors’ HRQOL. A study conducted shortly after the earthquake showed that the HRQOL of survivors is lower than that of the general population. With social support, this situation could be improved [26]. After the survivors received social intervention aimed at improving their HRQOL, their demand for health care services was reduced [27]. One study measured the HRQOL of survivors eight months and three years after the earthquake [28]. The HRQOL of survivors was still lower than the standard or below normal levels, especially in the Mental Component Summary (MCS), three years after the earthquake; however, the survivors could already be considered to have recovered eight months after the earthquake [28]. Given the frequency of earthquakes in the Sichuan area, this study assesses more factors of the survivors’ HRQOL in order to be able to address the health concerns of this affected community.

Specifically, we will develop a new perspective on the study of the health of earthquake survivors by assessing the effect of post-earthquake rescue policies on survivors’ HRQOL. In particular, we address the following questions. What is the current state of the survivors’ HRQOL? The government has given much support to the hard-hit areas; however, has the HRQOL of survivors improved? In the reconstruction, post-earthquake rescue policies play an important role. The government has rich resources to guide social forces to effectively perform reconstruction work; nevertheless, is the implementation of post-earthquake rescue policies effective? Can these policies improve the HRQOL of survivors? Are there any differences among the effects of different post-earthquake rescue policies on the reconstruction? To summarize the effects of reconstruction policies and assess the effects on the HRQOL of survivors, we need to know the answers to the questions above.

Can the post-earthquake rescue policies affect the HRQOL of earthquake survivors? A previous study found that the HRQOL of survivors has generally improved after receiving social support over time [26]. Thus, we propose the first hypothesis; Hypothesis 1: *The post-earthquake rescue policies can significantly improve the HRQOL of survivors.*

Can different post-earthquake rescue policies affect the HRQOL of survivors to the same degree? The degree of damage in all social areas caused by the earthquake differs. When the government develops these policies, they will follow the actual situation and make targeted policies. However, the government cannot guarantee the generation of similar strong effects. Thus, we propose the second hypothesis;

Hypothesis 2: *The effects of different post-earthquake rescue policies on the HRQOL of survivors have significant differences.*

We divided the HRQOL of survivors into two dimensions, namely physical component summary (PCS) and MCS. Is the degree of the effects of post-earthquake rescue policies on the two dimensions the same? What, then, is the degree of the impact of the mechanism? Thus, we propose the third hypothesis in this study;

Hypothesis 3: *The post-earthquake rescue policies directly affect the HRQOL of survivors.* The effect mechanism on the MCS is mainly achieved by improving the PCS.

Frequent earthquakes have constantly reminded us to pay more attention to the hard-hit disaster areas. The effect of policy implementation and the HRQOL of survivors need urgent attention. Based on these needs and research gaps, we have used the Medical Outcomes Study Short Form 36 (SF-36) to measure the HRQOL. Moreover, we measure the effects of the implementation of post-earthquake rescue policies for education, orphans, employment, poverty, legal, and social in five hard-hit disaster areas in Sichuan on the HRQOL of survivors. The study presents data on the effects of policy implementation and the HRQOL of survivors, analyzes the effects of different post-earthquake rescue policies on the HRQOL of survivors, and provides suggestions on how to improve the effects of reconstruction and the HRQOL of survivors for the government and non-governmental organizations.

## Methods

### Participants and procedure

Data were obtained through a survey in five counties in Sichuan in 2013 using a multi-stage sampling method. First, we selected five cities (Aba, Guangyuan, Deyang, Ya'an, and Chengdu) from 39 hard-hit disaster areas by using simple random sampling. Based on the same method, we then selected a county (Wenchuan, Qingchuan, Mianzhu, Lushan, and Dujiangyan) in each city as well as 400 randomly selected survivors in each county. Guided by investigators, each participant independently completed the questionnaire. A total of 2,000 questionnaires were distributed, and 1,672 were returned; the response rate was 83.6%.

### Instruments

The questionnaire asks about the post-earthquake rescue policies, cognition of survivors, and HRQOL of survivors. In this study, the measurements include the SF-36 scale and post-disaster rescue policy scale.

The SF-36 scale or the Medical Outcomes Study Short Form includes 36 items that measure eight domains and one self-assessed domain on health changes. In this study, the SF-36 measured the HRQOL of survivors after the earthquake and studied the effect of post-earthquake rescue policies on the HRQOL of survivors.

According to the “*National Master Plan on Post-Disaster Restoration and Reconstruction of Wenchuan Earthquake*” and other governmental policies, the post-disaster rescue policy scale consisted of the following six metrics:

1. ***Educational rescue policy***

The absorption of students in secondary vocational schools in disaster areas to the local school is encouraged. Local governments should expeditiously implement the policy that involves the education of the children of migrant workers into the public education system. The support of primary and secondary teachers’ allocation and training, especially for special education, should be increased. Financial assistance to students from economically disadvantaged families should be extended. University admission plans in disaster areas should also be expanded.

2. ***Orphan rescue policy***

The construction of facilities for social welfare, social relief, rehabilitation, etc., should be implemented. New public service sites should be configured for disabled facilities. Enterprises, social groups, and individuals are encouraged to offer a variety of support to orphans and disabled people.

3. ***Employment rescue policy***

People with employment difficulties due to disasters should be involved in the employment rescue coverage to ensure that each family has at least one earning member. Companies are encouraged to recruit urban workers unemployed due to disasters and for individual business operators to rise in the planned areas. The unemployment insurance rate of companies in the planned areas should be reduced as required. Social security subsidies, micro-lending, and other measures should promote employment.

4. ***Poverty rescue policy***

Investment should be increased in rural social security. The involvement of people who experience poverty due to disasters should receive at least a minimum living security system as required. Funds from the Rehabilitation Fund should be allocated for the reconstruction of the poor villages. Rehabilitation projects in ethnic minority areas and poor areas should not require the provincial local government to provide matching funds.

5. ***Legal rescue policy***

Legal rescue institutions at all levels should provide the survivors with legal advice, representation, criminal defense and other free legal services. Bar associations should provide necessary rescue for legal aid work. Judicial, administrative departments should oversee legal aid work.

6. ***Social security policy***

This policy should ensure that the work injury insurance of insured persons has been paid. Injured workers who are not insured should be assisted through public donations and the rescue system. The basic pension for enterprise retirees in the disaster areas should be paid. Companies that stopped production due to the disaster should not be penalized for holding social security payments. Unpaid pension insurance of the bankrupt enterprises after repayment could be written-off after approval.

### Analysis methods

#### Reliability analysis

Reliability refers to the consistency, stability, and reliability of survey results and indicates the degree of consistency of the results obtained by repeatedly measuring the same objects with the same methods. Reliability indicators are always indicated by correlation coefficients, which can be broadly divided into three categories: stability coefficients (cross-time consistency), equivalent coefficients (cross-form consistency), and internal consistency coefficients (cross-item consistency). Among them, the internal consistency coefficient is often used to measure the reliability of attitudes, opinions, and questionnaires. The internal consistency coefficient, that is, the Cronbach’s alpha coefficient, measures reliability by calculating the variance of each item and the total variance of all items.

The SF-36 scales and post-disaster assistance policy scales determine the HRQOL of survivors and their satisfaction with the policies, respectively. The questionnaires employed in this study utilize the aforementioned scales. In this study, we use the internal consistency coefficient to measure the homogeneity of items in the scales to measure the reliability of the questionnaire.

#### CFA model

The confirmatory factor analysis (CFA) model starts from the concept model, fits with the observation data and concept models, and tests the degree of support of the observation data to the concept model. The biggest advantage of the model is that it allows the testing of the relationship between observation variable and latent, and the latent variables under the situation in which there are measurement errors. Thus, the test effect is higher, and commonly used to study the model of social and psychological structures and their relationships.

#### Structural equation model

The structural equation model (SEM) is a linear statistical modeling technique that combines econometrics, sociology, psychology, and other statistical analysis measurement methods. It covers several original multivariate data analysis methods and applies nominal, ordinal, interval, and ratio variables. In empirical studies in management and social sciences, SEM is a major multivariate data analysis method along with multiple regression analysis method. SEM is used to explain the relationship between multiple variables and concepts. Statistically, by looking for some variables that can be observed as a potential variable “identity,” we could analyze the relationships between the “latent variables”.

In this study, we identified the internal relationships between the latent variables with the CFA model and established a suitable SEM based on it to study the effects of different policies on the HRQOL of survivors.

## Results

### Basic statistics of policy and SF-36 scales

Table 1 shows the basic statistics. In the items regarding health, the higher scores indicate a greater HRQOL of the respondents. In the items regarding policies, higher scores indicate a higher satisfaction with the policies among the survivors.

**Table 1:** SF-36 items basic statistics

**Domains**	**Mean value**	**Variance**	**Minimum**	**Maximum**	**Range of values**
Physical functioning	44.84	594.21	0	100	[1,100]
Role physical	43.20	1049.21	0	100	[1,100]
Bodily pain	45.88	700.24	0	90	[1,100]
General health	31.50	362.42	0	80	[1,100]
Vitality	41.26	635.54	0	100	[1,100]
Social functioning	39.76	769.93	0	100	[1,100]
Role emotional	44.35	1262.81	0	100	[1,100]
Mental health	39.35	606.76	0	96	[1,100]
Educational rescue policy	2.46	1.95	1	5	[1,5]
Orphan rescue policy	2.38	1.87	1	5	[1,5]
Employment rescue policy	2.53	1.85	1	5	[1,5]
Poverty rescue policy	2.41	1.87	1	5	[1,5]
Social security policy	2.60	1.64	1	5	[1,5]
Legal rescue policy	2.58	1.70	1	5	[1,5]

In the eight domains of HRQOL, the mean value was lower than 50, which means the HRQOL of survivors is not good. Among them, physical functioning (PF), the mean values of four items in role physical (RP), role emotional (RE), and social functioning (SF) were relatively low. This finding indicates the HRQOL on these four domains was worse. The mean values of six items on the satisfaction with post-earthquake rescue policies were lower than 2, which reflect the low satisfaction of survivors regarding the policies. Thus, the post-earthquake rescue policies need to be further improved.

### SF-36 reliability analysis

**Figure 2:**
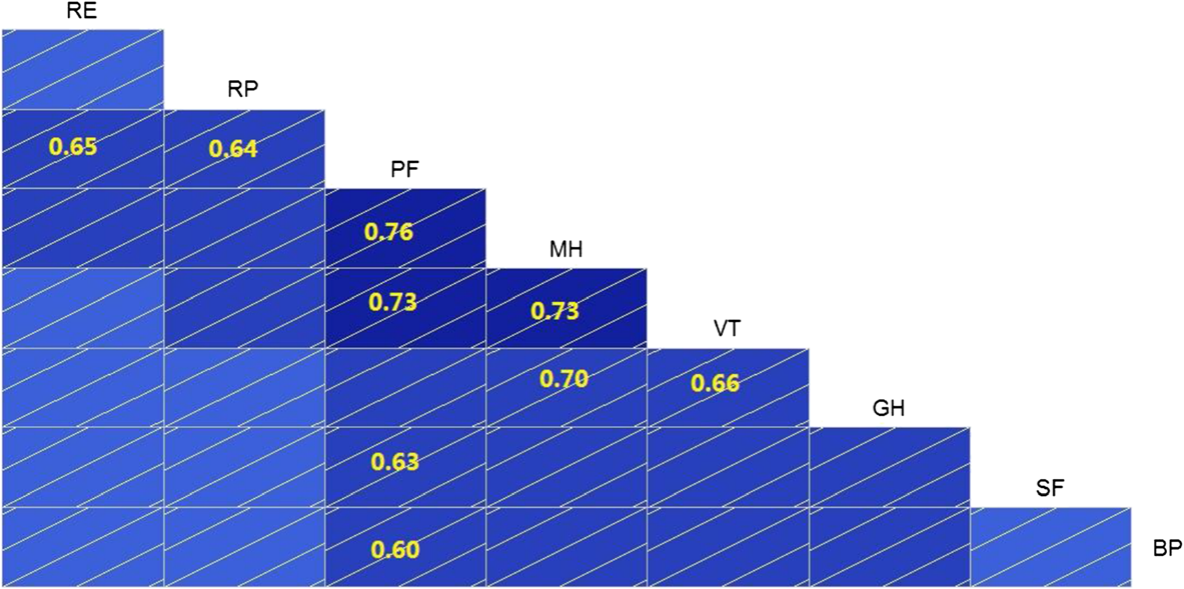
Correlation coefficient of eight domains.

The total Cronbach’s alpha coefficient of the SF-36 scale is 0.913. The general evaluation criterion of reliability is the range scale of reliability of over 5.0. When the Cronbach’s alpha coefficient is over 0.70, the total reliability is very good. In other words, the survey topics had strong internal consistency. Table 2 and Figure 2 show that the SF-36 scale was very reliable in measuring the HRQOL of survivors. Figure 2 indicates the correlation coefficients which are greater than 0.6. Each alpha coefficient in the eight domains was over 0.80 (Table 2), which means very good internal consistency of the scale.

**Table 2:** Policy-SF-36 Cronbach’s alpha coefficient

**Items**	**Cronbach’s alpha if items were deleted**
Physical functioning	0.845
Mental health	0.847
Vitality	0.849
Role physical	0.858
General health	0.853
Bodily pain	0.857
Role emotional	0.862
Social functioning	0.856
Educational rescue policy	0.871
Orphan rescue policy	0.871
Employment rescue policy	0.871
Poverty rescue policy	0.871
Social security policy	0.872
Legal rescue policy	0.872

### CFA Model results

We established the corresponding CFA models and discussed their validity. Considering the actual needs, the CFA results often require overall model fitting to evaluate the fitting degree between model and data. Overall model fitting employs several aspects. First, the absolute goodness of fit is used to predict to what extent the model can determine the covariance matrix and relevant matrix. Chi-square value (*χ*^2^), chi-square value/degrees of freedom (*χ*^2^/*df*), and goodness-of-fit index (GFI), among others, are utilized in this model. Second, the added goodness of fit is used to compare the theoretical model and virtual model. The increasing fit index (IFI) is an example of indices used in this model.

The *χ*^2^ test investigates the assumption that the covariance matrix of the selected model matches the covariance matrix of the observed data. The original assumption is that the covariance matrix of the selected model is equal to that of the sample. If the model is a good fit, then *χ*^2^ should not be significant. In this case, the bad fitting model is rejected. GFI, IFI, and comparative fit index (CFI) are relatively fit indices; their values lie between 0 and 1. Theoretically, these indices can produce meaningless negative values. If their value is close to 1, then the model has a good fit. The model is acceptable if the value of these indices is greater than 0.90. The closer their value is to 1, the better the fit of the model.

**Figure 3:**
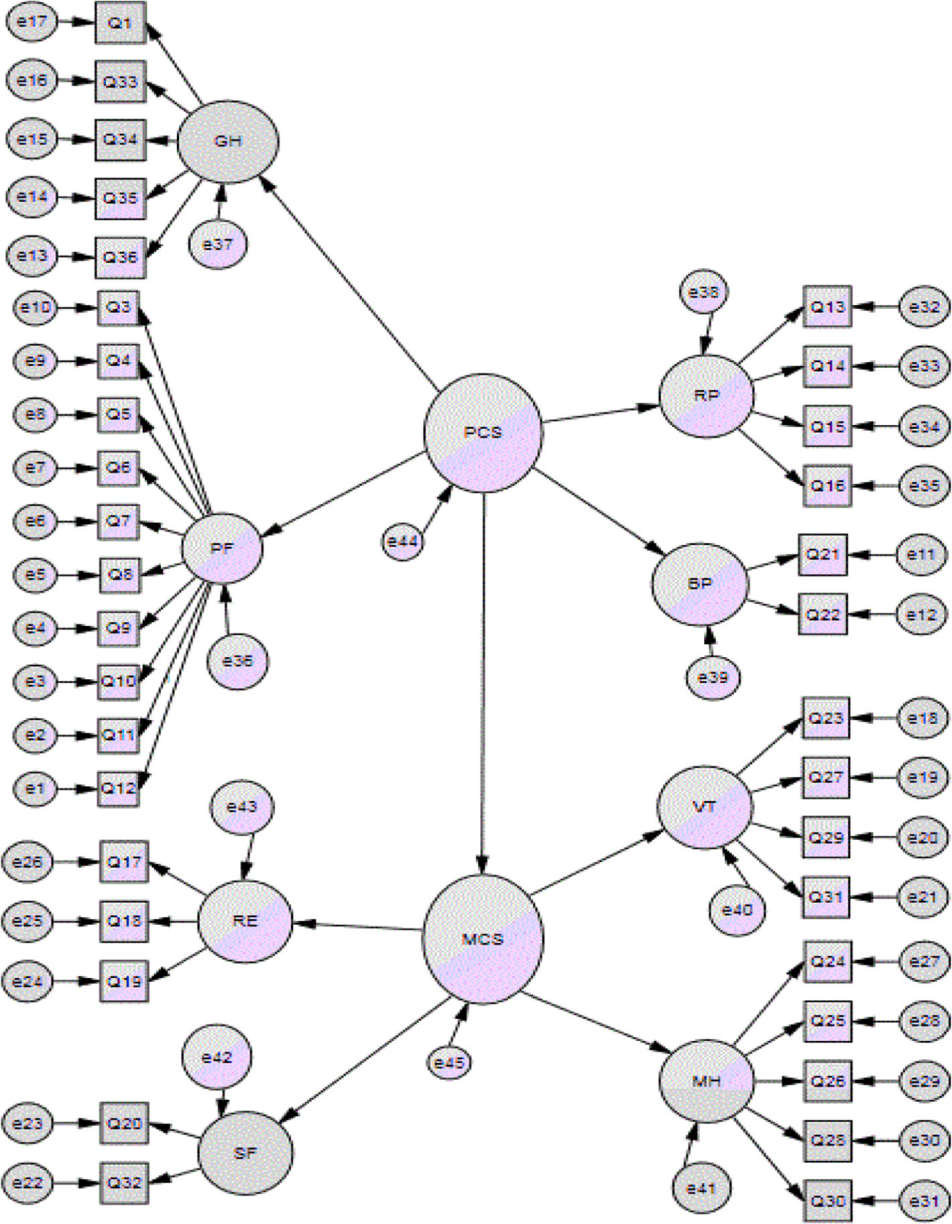
CFA model for SF-36 scale.

The SF-36 scale consists of eight domains, which represent the PCS and MCS. Hence, we established a CFA model to study the interrelationship between the MCS and PCS (Figure 3).

The result shows that *χ*^2^/*df* = 2.14, CFI = 0.963, IFI = 0.963. As shown in Figure 3 and Table 3, the PCS directly affected the MCS. Hence, health domains of the PCS and MCS had a significantly positive correlation with each health factor, which matches the reality. The figure also indicated that the PCS has a positive correlation with the PF, RP, bodily pain (BP), and general health (GH). The MCS had a positive correlation with vitality (VT), SF, RE, and mental health (MH).

**Table 3:** CFA model results

	**Estimate**	**SE**	**C.R.**	***P***		**Estimate**	**SE**	**C.R.**	***P***
PCS- > MCS	4.212	0.239	17.647	***	GH- > Q35	1.319	0.098	13.438	***
PCS- > PF	1.702	0.101	16.843	***	GH- > Q34	1.474	0.104	14.174	***
PCS- > GH	2.233	0.166	13.458	***	GH- > Q33	1.658	0.114	14.528	***
PCS- > RP	1.000				GH- > Q1	1.587	0.110	14.380	***
PCS- > BP	3.916	0.230	17.032	***	VT- > Q23	1.000			
MCS- > VT	1.000	0.050	21.467	***	VT- > Q27	1.060	0.053	20.035	***
MCS- > MH	1.073	0.038	19.553	***	VT- > Q29	0.876	0.048	18.423	***
MCS- > SF	0.752	0.014	18.843	***	VT- > Q31	1.102	0.050	21.970	***
MCS- > RE	0.258				SF- > Q32	1.000			
PF- > Q12	1.000	0.059	17.827	***	SF- > Q20	0.510	0.030	17.205	***
PF- > Q11	1.056	0.059	18.636	***	RE- > Q19	1.000			
PF- > Q10	1.097				RE- > Q18	0.947	0.055	17.345	***
PF- > Q9	0.975	0.057	17.110	***	RE- > Q17	0.855	0.053	15.991	***
PF- > Q8	1.066	0.057	18.765	***	MH- > Q24	1.000			
PF- > Q7	1.096	0.057	19.342	***	MH- > Q25	1.007	0.042	23.863	***
PF- > Q6	1.099	0.058	19.068	***	MH- > Q26	1.056	0.045	23.286	***
PF- > Q5	1.002	0.055	18.324	***	MH- > Q28	0.868	0.039	22.041	***
PF- > Q4	0.945	0.053	17.753	***	MH- > Q30	0.864	0.045	19.321	***
PF- > Q3	0.989	0.057	17.419	***	RP- > Q13	1.000			
BP- > Q21	1.000				RP- > Q14	0.785	0.057	13.769	***
BP- > Q22	0.900	0.047	19.041	***	RP- > Q15	1.006	0.061	16.493	***
GH- > Q36	1.000				RP- > Q16	0.930	0.060	15.468	***

Note: *** indicates the path coefficients are significant when their significance is over 99%. PF: Physical functioning; RP: Role physical; BP: Bodily pain; GH: General health; VT: Vitality; SF: Social functioning; RE: Role emotional; MH: Mental health.

### SEM model results

**Figure 4:**
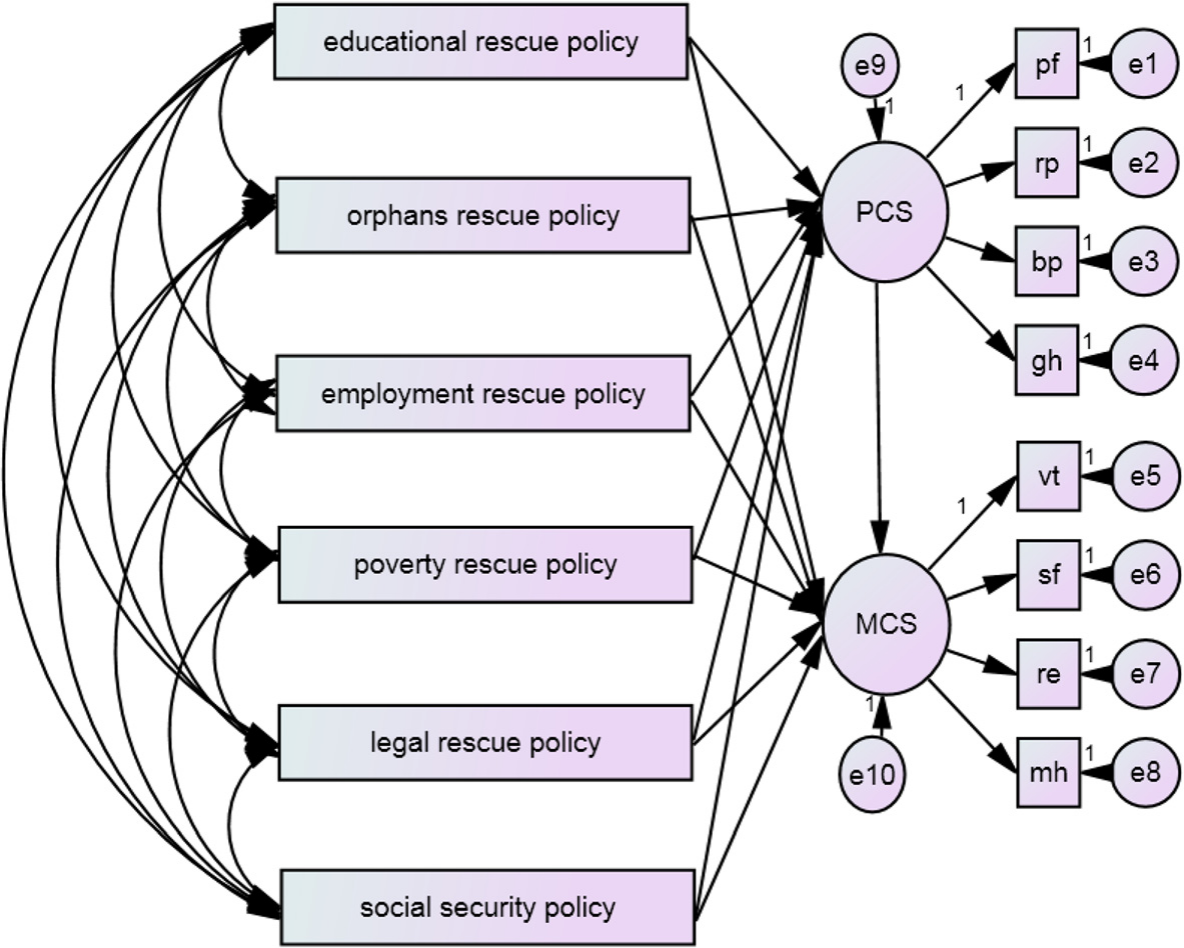
SEM model of effect of post-earthquake rescue policies on HRQOL of survivors.

We modified the SEM model and obtained the best model fitting results. The results indicated *χ*^2^ = 110.3, GFI = 0.991, *χ*^2^/*df* = 2.00, *P* <0.05 (Table 4). The GFI, CFI, and IFI values were large and close to 1. Therefore, the model fitting was relatively good. Generally, different post-earthquake rescue policies influenced the HRQOL of survivors. We compared the differences among the effects of each post-disaster rescue policy on the HRQOL of survivors through the model results. We found all path coefficients were significant (Figure 4).

**Table 4:** SEM model fit indexes

**Model fit index**	***χ***^2^	***df***	***χ***^2^/*df*	**GFI**	**CFI**	**IFI**	***P***
Value	110.3	55	2.00	0.991	0.996	0.996	0.000

As we can see from the Figure 4, post-earthquake rescue policies have significant positive effects on the promotion of the HRQOL of survivors. However, different policies have resulted in different effects. Table 5 shows the path coefficients and corresponding statistics of policies on two health factors. To measure whether each path coefficient is significant or not, the CR value constructed by the Z statistics should be used. Each CR value corresponds to a *P* value. *P* value reflects whether the path coefficients are statistically significant or not. The greater the *P* value is, the more significant the corresponding path coefficient is. Conversely, if the *P* value is smaller, then the corresponding path coefficient is less significant.

**Table 5:** SEM model results

	**Estimated value**	**SE**	**C.R.**	***P***
PCS < −Educational rescue policy	1.938	0.287	6.743	***
PCS < −Orphan rescue policy	2.063	0.305	6.767	***
PCS < −Employment rescue policy	2.702	0.296	9.138	***
PCS < −Poverty rescue policy	1.973	0.309	6.390	***
PCS < −Social security policy	4.325	0.345	12.550	***
PCS < −Legal rescue policy	5.201	0.327	15.899	***
MCS < −Educational rescue policy	0.824	0.270	3.052	0.002
MCS < −Orphan rescue policy	0.297	0.286	1.038	0.299
MCS < −Employment rescue policy	−0.079	0.289	−0.272	0.786
MCS < −Poverty rescue policy	−0.325	0.288	−1.127	0.260
MCS < −Social security policy	0.458	0.361	1.269	0.204
MCS < −Legal rescue policy	−0.244	0.370	−0.658	0.511

Note: *** indicates the path coefficients are significant when their significance is over 99%.

From the SEM model results, we know that some *P* values were relatively high, which means that some path coefficients were unable to pass the test. However, only coefficients that passed the test can explain the corresponding correlation. If the passed path coefficients are all positive, the policies they correspond to have a significant effect on promoting the HRQOL of survivors. For example, the effect of the educational rescue policy on PCS passed the test; its estimated value is 1.938, which means the educational rescue policy has a significant effect on promoting the PCS.

From the results in Table 5, the following can be observed. The educational rescue policy directly affects the physical and mental health of survivors. As the model results show, the path coefficients of educational rescue policy on the PCS and MCS passed the significance test, and both are positive. The orphan rescue policy directly affects the PCS of survivors. The path coefficient of the orphan rescue policy on the PCS is significant and positive, which means the policy positively affects the PCS of survivors. The poverty rescue policy also has a significant effect on promoting the PCS of survivors. Moreover, this policy indirectly affects the MCS mainly through the PCS. The employment rescue policy directly affects the PCS of survivors. The path coefficient of employment rescue policy on the PCS is significant and positive, which means the employment rescue policy positively affects the PCS of survivors. Compared with the preceding three kinds of policies, the employment rescue policy is different from the orphan and poverty rescue policies. The employment rescue policy has a greater effect on promoting the PCS of survivors. Both the social security and legal rescue policies directly affect the PCS of survivors, although the effect on the MCS is not significant. Two policies have the greatest significance on the PCS. The path coefficient of legal rescue policy on the PCS is 5.201, which is obviously higher than that of other five policies. Finally, we should pay attention to the above policies that have indirect effects on the MCS of survivors, mainly through the PCS. As shown in Table 6, only one out of the six kinds of policies, the educational rescue policy, passed the test. Hence, only the educational rescue policy significantly and directly affects the MCS of survivors.

## Discussion

The Sichuan province is an earthquake-prone area. Earthquake survivors have been suffering from the effects of the disasters, and their HRQOL needs our attention. Therefore, despite the long-term nature of the reconstruction task, the effects of implementation of rescue policies needs to be assessed now. By measuring and establishing the above described models, our main findings are as follows:

1. In this study we develop a new perspective to study the health of survivors. We tried to the study the effect of post-earthquake rescue policies on the survivors’ HRQOL.

2. We calculated the total Cronbach’s alpha coefficient of post-earthquake rescue policies and SF-36 scales as well as each of their dimensions. The reliability of both the two scales is relatively good. The scales have good internal consistency.

3. As the basic statistics of policy and SF-36 scales show, the general HRQOL of survivors is not good. They have low satisfaction with the post-earthquake rescue policies.

4. We established a CFA model to study the relationship between the MCS and PCS. The result shows that the PCS directly affected the MCS. Hence, health domains of the PCS and MCS had a significantly positive correlation with each health factor. The CFA model also indicated that the PCS has a positive correlation with the PF, RP, BP, and GH. The MCS had a positive correlation with VT, SF, RE, and MH.

5. We studied the effect of different post-earthquake rescue policies on the HRQOL of survivors by using the SEM model. Every fit index showed that the model fitting is good, and most of indexes have met acceptable standards. We obtained the following features of post-earthquake rescue policies on the HRQOL of survivors:

i) The post-earthquake rescue policies can significantly improve the HRQOL of survivors. The six policies have direct effects on promoting the PCS of survivors. The first hypothesis was mainly supported.

ii) The impact mechanism of the six policies on the MCS of survivors is basically the same. Aside from the educational rescue policy, other policies affected the MCS indirectly through the PCS. The third hypothesis was mainly supported. In other words, the post-earthquake rescue policies directly affected the PCS of survivors. The policies affected the MCS indirectly through the PCS.

iii) Differences between the path coefficients in the model are relatively large, which means the differences in the effects of the policies on the HRQOL of survivors are relatively large. Among them, the legal rescue and social security policies are most effective on the HRQOL of survivors. The education, orphan, employment, and poverty rescue policies have relatively weak effects on the recovery of the HRQOL of survivors. The second hypothesis was mainly supported.

The government plays the crucial roles of being a leader and a guide. The implementation of reasonable policies can positively affect the promotion of the reconstruction effort. Among them, the government has provided different medical services in various phases of the reconstruction. Before 31 December 2008, the individuals who were injured because of the earthquake received free medical aid. The normal health care system has been made appropriate to the survivors in the hard-hit disaster areas. In fact, the survivors had high satisfaction with the medical services arranged by the government [29]. The survivors welcomed the post-disaster emergency medical policy. However, medical services focused on the physical health of survivors and ignored their mental health. The medical policy of the government should pay more attention to the mental health of survivors.

Shortly after the earthquake, the most pressing needs of survivors included housing, employment, and education. However, firstly, with the reconstruction moving forward, legal and social security issues may become more important. Because of land requisition, the number of farmers who lost their land increased. In terms of land use, they need urgent legal services. Secondly, while the current level of compensation for land requisition in China is low, the way to rehouse the survivors is simple and straightforward, without securing their long-term livelihoods [30]. The landless farmers also lack support from the social security system. Hence, the legal and social security rescue policies provided by the government solved their immediate needs. The program truly met the demands and improved the HRQOL of survivors. Lastly, the government adhered to implementing the education, orphans, employment, and poverty rescue policies after every natural disaster. Nevertheless, their effects on promoting the HRQOL of survivors were relatively weak.

This finding prompts us to propose that post-earthquake rescue policies should be adjusted to ensure a better reconstruction effect. Government services should uphold the “people-oriented” concept and should help citizens to articulate and achieve common interests. The HRQOL of survivors has improved, but they have greater needs, such as a better social security system and more professional legal services. Therefore, our government should ensure the implementation of all six policies that can favor legal rescue and social security policies. During policy implementation, a good implementation of mechanisms in monitoring, effect assessment, and feedback should be established among survivors, policy makers, and implementers to ensure full exchange and communication. Some duplicated or rigid policies should be terminated or adjusted timely.

Surely, this study has some limitations. First, we selected only five prefecture-level cities in the Sichuan province in our sample. Sichuan earthquakes not only affected cities of the Sichuan province but also villages of nearby provinces, such as Gansu and Shaanxi; future studies should be extended to cover the said areas. Second, this study focused only on post-earthquake rescue policies. Other policies may also affect the HRQOL of survivors. Future studies could focus on other post-disaster reconstruction policies of the government. Lastly, concern on the HRQOL of survivors should last. Future studies can do follow-up investigation on the HRQOL of survivors.

## Conclusions

Many studies on the health of earthquake survivors have focused on the prevalence rate of mental disorders and their risk factors. By contrast, the present study investigates the health of earthquake survivors from the perspective of HRQOL. Among the few studies about HRQOL, most have focused the positive effect of social support on survivors’ HRQOL. Furthermore, there is a research gap on the effect of government support policies. Therefore, we developed a new perspective to study the health of survivors. We provide an assessment of post-disaster rescue policies by survivors in hard-hit disaster areas and renew interest in the HRQOL of these survivors, as well as the effect of post-earthquake rescue policies on the survivors’ HRQOL. Our main findings are as follows:

1. As the basic statistics of policy and SF-36 scales show, the general HRQOL of survivors is not good; they have low satisfaction with the post-earthquake rescue policies.

2. The post-earthquake rescue policies can significantly improve the HRQOL of survivors. The six policies have a direct effect on promoting the PCS of survivors.

3. The impact mechanism of the six policies on the MCS of survivors is basically the same. Aside from the educational rescue policy, other policies affect the MCS indirectly through the PCS.

4. Differences between the path coefficients in the model are relatively large. This finding indicates relatively large differences in the effects of different policies on the HRQOL of survivors. Among these policies, legal and social security are the most effective on the HRQOL of survivors. The education, orphan, employment, and poverty rescue policies have relatively weak effects on the recovery of the HRQOL of survivors.

We finally know the effect of post-earthquake rescue policies on the HRQOL of survivors. To enhance the positive effect, the implementation of policies should be changed and adjusted to the actual needs of survivors. A related medical rescue policy should focus more on the mental health of survivors.

## Endnote

^a^This photo was taken in Guangyuan, one of the sites where the questionnaires were distributed.

## Abbreviations

BP: Bodily pain; CFA: Confirmatory factor analysis; GH: General health; HRQOL: Health-related quality of life; MCS: Medical component summary; MH: Mental health; PCS: Physical component summary; PF: Physical functioning; SE: Standard error; SEM: Structural equation model; SF: Social functioning; SF-36: Medical Outcomes Study Short Form 36; RE: Role emotional; RP: Role physical; VT: Vitality.

## Competing interests

The authors declare that they have no competing interests.

## Authors’ contributions

YL wrote and revised the manuscript, was responsible for the design of the study, and performed the statistical analysis. XW participated in the statistical analysis. Both authors read and approved the final manuscript.
